# Efficacy and safety of remimazolam tosylate for sedation during upper gastrointestinal endoscopy: study protocol for a multicenter randomized controlled trial

**DOI:** 10.1186/s13063-022-06935-0

**Published:** 2022-12-12

**Authors:** Huichen Zhu, Zhongxue Su, Xiaorong Huai, Caiyang Chen, Xiao Zhang, Jie Zhou, Diansan Su

**Affiliations:** Department of Anesthesiology, Renji Hospital, School of Medicine, Shanghai Jiaotong University School of Medicine, 160 Pujian Road, Shanghai, 200127 China

**Keywords:** Remimazolam tosylate, Efficacy, Safety, Sedation, Upper gastrointestinal endoscopy

## Abstract

**Background:**

Gastrointestinal endoscopy has been associated with difficult experiences and can leave patients with an unpleasant impression. Propofol and midazolam are the most commonly used intravenous anesthetics for sedation during gastrointestinal endoscopy. However, cardiac and pulmonary adverse events are the primary concerns associated with the use of these sedatives. Remimazolam tosylate is an ultra-short-acting benzodiazepine drug with a mild inhibitory effect on the respiratory and circulatory systems. These properties qualify remimazolam tosylate to be used as a replacement for propofol or midazolam as a sedative during gastrointestinal endoscopy. This study aims to describe the efficacy and safety of remimazolam tosylate as a sedative for upper gastrointestinal endoscopy.

**Methods:**

A multicenter, randomized, single-blind, parallel-controlled, noninferiority clinical study will be conducted to evaluate the efficacy and safety of remimazolam tosylate as a sedative during upper gastrointestinal endoscopy. Participants (*n* = 1800) will be randomized to receive remimazolam tosylate at 0.15 mg/kg (experimental group 1), remimazolam tosylate at 0.2 mg/kg (experimental group 2), or propofol at 1.5 mg/kg (control group). Procedure success will be assessed and defined as the completion of upper gastrointestinal endoscopy without the administration of a rescue sedative agent or more than two top-up doses of the trial drug in any 5-min period after initial administration. Sedation quality will be evaluated using the Modified Observer’s Assessment of Alertness/Sedation score. Adverse events will be recorded to evaluate safety.

**Discussion:**

This study will determine the optimal dosage of remimazolam tosylate during upper gastrointestinal endoscopy and will describe its efficacy and safety. These findings may contribute to a more comfortable and safer experience for patients compared with that when the conventional sedative propofol is used.

**Trial registration:**

ClinicalTrials.gov NCT04727034. Registered on February 18, 2021.

**Supplementary Information:**

The online version contains supplementary material available at 10.1186/s13063-022-06935-0.

## Background

In current clinical practices, midazolam or propofol are the main anesthetic sedatives commonly used during outpatient procedures, such as gastrointestinal endoscopic diagnosis and treatment [1]. Propofol has certain advantages, including rapid onset of action, short recovery profile, antiemetic properties, equivalent amnesic effects, and patient’s comfort [2]. Therefore, propofol is frequently used as a sedative agent during standard endoscopic procedures worldwide [3]. However, it has a disadvantage in the form of a narrow therapeutic index, which potentially causes cardiovascular and respiratory depression and hypoxia, resulting in the potential need for urgent endotracheal intubation [4]. Supplemental oxygen is often necessary when propofol is used. Nonanesthesia specialists have not been authorized to use propofol in many countries, resulting in patients not benefiting from propofol.

Midazolam is a commonly used sedative during gastrointestinal endoscopy because of the lesser risk of complication and medical legal issues [5]. Midazolam metabolites, namely, 1-hydroxymidazolam and α-hydroxymidazolam, also show pharmacodynamic potential comparable to midazolam [6, 7]. There is a concern regarding the potential for repeat sedation when the active metabolite becomes bioavailable [8].

Remimazolam is a new type of narcotic and sedative that belongs to the same benzodiazepine class as midazolam [9]. Remimazolam, an ester-based drug, is designed to be rapidly hydrolyzed in the body by ubiquitous tissue esterases to an inactive carboxylic acid metabolite (CNS 7054) [10]. It is broken down to produce metabolites with significantly weakened affinity for type A gamma-aminobutyric acid (GABAA) receptors [11]. It is characterized by a more rapid offset and quicker return to normal cognitive and memory function than that of midazolam [12, 13]. Compared with propofol, remimazolam has no injection pain and has a lower incidence of hypotension [14]. In the USA, only anesthesiologists are authorized to administer propofol [15]. However, unlike propofol, remimazolam belongs to benzodiazepine, which allows it to be used without specific restrictions. Therefore, remimazolam has good application prospects in upper gastrointestinal endoscopy.

Remimazolam tosylate (RT), which acts on GABA receptors, is a new ultra-short-acting benzodiazepine [16] developed by HengRui Medicine Co., Ltd., China. Similar with remimazolam, RT has a short half-life that results in quick-acting onset and recovery compared with currently available short-acting sedatives [17]. Recent studies have reported that RT is suitable for short operations, such as gastrointestinal endoscopy, hysteroscopy, bronchoscopy, and closed reductions of long-bone fractures [18].

We will perform this randomized, single-blind, parallel-controlled, noninferiority clinical trial at five centers. Therefore, this study aims to determine the optimal dosage of remimazolam tosylate during upper gastrointestinal endoscopy and to describe its efficacy and safety in Chinese patients undergoing upper gastrointestinal endoscopy.

### Aims and objectives

Compared with propofol, the safety and efficacy of remimazolam tosylate during upper gastrointestinal endoscopy are verified based on the completion of upper gastrointestinal endoscopy, vital signs of participants, and satisfaction of endoscopists and patients.

### Trial design

A multicenter, randomized, single-blind, parallel-controlled, noninferiority study protocol was formulated. We will compare the efficacy and safety of remimazolam tosylate with those of propofol during sedation for upper gastrointestinal endoscopy. This clinical trial has been approved and is supported by the ethics committee of Renji Hospital, Shanghai Jiaotong University School of Medicine (KY2020-127).

This study was presented according to the recommendations of the Standard Protocol Items: Recommendations for Interventional Trials (SPIRIT) (Supplemental file, SPIRIT checklist). This trial was registered on 18 February 2021 in ClinicalTrials.gov, NCT04727034. The trial registration dataset is presented in the Supplemental table.

## Methods

### Participants, interventions, and outcomes

#### Study setting

This study will recruit 1800 participants from Renji Hospital, Shanghai Jiaotong University School of Medicine, Shanghai East Hospital, The First Affiliated Hospital of Nanchang University, The First Affiliated Hospital of Jiaxing University, and The Second Affiliated Hospital of Jiaxing University.

#### Eligibility criteria

Participants will be recruited primarily from the abovementioned five centers. Table 1 presents a summary of the inclusion and exclusion criteria.

**Table 1:** Inclusion/exclusion criteria

Inclusion criteria	Exclusion criteria
(1) Age, ≤18 and ≤60 years, no gender limit	(1) Need to perform complicated endoscopic techniques for diagnosis and treatment, such as cholangiopancreatography surgery, endoscopic ultrasonography, endoscopic mucosal resection, endoscopic submucosa stripping, and oral endoscopic muscle dissection
(2) Undergoing routine upper gastrointestinal endoscopic diagnosis and treatment	(2) Intend to undergo tracheal intubation
(3) American Society of Anaeshesiologists (ASA) classification I–II	(3) Judged to have difficulty in managing the respiratory tract (modified Mallampati score is IV)
(4) 18 kg/m^2^ < body mass index (BMI) < 28 kg/m^2^	(4) Anemia or thrombocytopenia, (hemoglobin < 90 g/L, platelet count <80 × 10^9^/L)
(5) Time of upper gastrointestinal endoscopy not exceeding 30 min	(5) Diagnosed with lung diseases (asthma, bronchitis, chronic obstructive pulmonary diseases, pulmonary bullae, pulmonary embolism, pulmonary edema, and lung cancer)
(6) Clearly understand and voluntarily participate in the study; provide signed informed consent	(6) Diagnosed with liver and kidney diseases (aspartate aminotransferase and/or alanine aminotransferase ≥2.5 × upper limits of normal (ULN), total bilirubin ≥1.5 × ULN, and blood creatinine levels greater than the upper normal limit)
	(7) History of drug and/or alcohol abuse within 2 years before initiating the screening period; average daily alcohol consumption of >2 units of alcohol (1 unit = 360 mL beer or 45 mL liquor with 40% alcohol content or 150 mL grapes liquor)
	(8) Blood pressure not satisfactorily controlled by antihypertensive drugs (sitting systolic blood pressure, ≥160 mmHg during the screening period and/or diastolic pressure during the screening period pressure, ≥100 mmHg)
	(9) Sitting systolic blood pressure of ≤90 mmHg during the screening period
	(10) Pregnant or breastfeeding
	(11) Allergies or contraindication to benzodiazepines, opioids, propofol, and lidocaine
	(12) Participated in other drug clinical trials in the past 3 months
	(13) Investigator’s judgment as an unsuitable participant
	(14) Diagnosed with heart disease (heart failure, angina pectoris, myocardial infarction, and heart rhythm abnormalities)

#### Recruitment and informed consent

Participants who are scheduled to undergo sedated upper gastrointestinal endoscopy in the outpatient gastrointestinal endoscopy operating room will be included in this study. They will be given an informed consent form, and they will be given the opportunity to review the consent and ask questions regarding the study. Interested participants will be screened using the inclusion and exclusion criteria. Eligible participants will sign an informed consent form. Study participation is voluntary, and the participants can withdraw from the study at any point of time. The recruitment and consent of the study participants by the members of the research team are in accordance with the Good Clinical Practice (GCP) guidelines. During the clinical trial, researchers will immediately report any serious adverse events occurring in the participants to the director-in-charge of the clinical trial of the research institution and will contact Professor Diansan Su or Dr. Huichen Zhu.

#### Allocation

Participants will be randomly assigned to one of the following three groups: experimental group 1 (remimazolam tosylate 0.15 mg/kg), experimental group 2 (remimazolam tosylate 0.2 mg/kg), or control group (propofol 1.5 mg/kg). A central randomization system will be used to randomize the participants. The system is stratified by each center, and the block length is six. Using the allocation sequence of the central randomization system, participants will be randomly assigned in a 1:1:1 ratio to experimental group 1, experimental group 2, or control group. Eligible participants will receive a unique random number upon enrollment. If multiple participants are scheduled on the same day, randomization will be performed based on the time of the participants’ arrival rather than the sequence of screening.

#### Procedure of the trial

Routine preparations are conducted before upper gastrointestinal endoscopy (generally fasting for at least 6 h preoperatively and no drinking water for at least 2 h). Before inducing sedation for upper gastrointestinal endoscopy, venous access will be established. During the procedure, peripheral oxygen saturation (SpO_2_), heart rate, continuous right upper limb noninvasive blood pressure, and Modified Observer’s Assessment of Alertness/Sedation (MOAA/S) score will be monitored. Specific monitoring indicators are presented in Table 2. Perioperative respiratory-related adverse events and participants’ treatment methods are shown in Table 3, and adverse events of anesthesia and sedation are shown in Table 4.

**Table 2:** The specific monitoring indicators

Monitoring indicators	Stage	Monitoring frequency
SpO_2_, heart rate, blood pressure, MOAA/S score	Before beginning sedation induction	After taking lidocaine hydrochloride mucilage and entering the room, the patient is placed in the left decubitus position on the examination bed, and indicators are recorded:
	Sedation induction started when fully awake	(1) The initial dose of remimazolam or propofol is intravenously administered (this time will be recorded as 0 min) (2) Within 1 min after the initial intravenous dose (3) When the upper gastrointestinal endoscope is successfully inserted (4) After successfully inserting the upper gastrointestinal endoscope, every 3 min until the end of upper gastrointestinal endoscopy (5) At the end of upper gastrointestinal endoscopy (6) When the patient is fully alert (MOAA/S score of 5 points for three consecutive times; for an MOAA/S score of 5, it should reach 5 the next two times).

**Table 3:** Perioperative respiratory-related adverse events and treatment records

SpO_2_ before the procedure (no oxygen)		Lowest SpO_2_ during the procedure		Is SpO_2_ <90% during the procedure?	Yes □ No□
Duration of SpO_2_ <90%		Did oxygen flow increase during the procedure?	Yes □ No □	Is the patient's mandible lifted?	Yes □ No□
Is mask ventilation required?	Yes□ No□			Is endotracheal intubation performed?	Yes □ No □

Treatment of respiratory-related adverse events: When SpO_2_ is <95%, the participant will be lifted to the mandible. If SpO_2_ is <90%, the oxygen flow of the nasal catheter will be increased to 6L/min. If SpO_2_ is still <90%, the procedure will be stopped, the upper gastrointestinal endoscope will be pulled out, and positive pressure ventilation will be performed using a mask. If SpO_2_ cannot be corrected, laryngeal mask airway or tracheal intubation will be performed to control breathing

**Table 4:** Adverse events of anesthesia and sedation

Step 1: Was there one or more adverse events associated with this sedation encounter?				
No, this form is now complete.	Yes, fill out remainder of form below.			
Step 2: Please DESCRIBE the adverse events(s). Check all that apply.				
Minimal risk descriptors	Minor risk descriptors		Sentinel risk descriptors	
Vomiting/Retching	Oxygen desaturation (75–90%) for <60s		Oxygen desaturation, severe (<75% at any time) or prolonged (<90% for >60s)	Other, specify below
Subclinical respiratory depression^a^	Apnea, not prolonged		Apnea, prolonged (>60 s)	
Muscle rigidity, myoclonus	Airway obstruction		Cardiovascular collapse/shock^g^	
Hypersalivation	Failed sedation^e^		Cardiac arrest/absent pulse	
Paradoxical response^b^	Allergic reaction without anaphylaxis			
Recovery agitation^c^	Bradycardia^f^			
Prolonged recovery^d^	Tachycardia^f^			
	Hypotension^f^			
	Hypertension^f^			
	Seizure			
Step 3: Please note the INTERVENTIONS performed to treat the adverse events(s). Check all that apply.				
Minimal risk	Minor risk	Moderate risk	Sentinel intervention	
No intervention performed	Airway repositioning	Bag valve mask-assisted ventilation	Chest compressions	Other, specify below
Administration of:	Tactile stimulation	Laryngeal mask airway	Tracheal intubation	
Additional sedative(s)	or the administration of:	Oral/nasal airway	or the administration of:	
Antiemetic	Supplemental oxygen, new or increased	Continuous positive airway pressure (CPAP)	Neuromuscular block	
Antihistamine	Antisialogogue	or the administration of:	Pressor/epinephrine	
		Reversal agents	Atropine to treat bradycardia	
		Rapid i.v. fluids		
		Anticonvulsant i.v.		
Step 4: Please note the OUTCOME of the adverse events(s). Check all that apply.				
Minimal risk outcome	Moderate risk outcome		Sentinel outcome	
No adverse outcome	Unplanned hospitalization or escalation of care^h^		Death	Other, specify below
			Permanent neurological deficit	
			Pulmonary aspiration syndrome^i^	
Step 5: Assign a SEVERITY rating to the adverse event(s) associated with this sedation encounter.				
If there are any options checked in the Sentinel columns above, then this is a Sentinel^j^ adverse event.				
If the most serious option(s) checked above are Moderate risk, then this is a Moderate^k^ risk adverse event.				
If the most serious option(s) checked above are Minor risk, then this is a Minor^l^ risk adverse event.				
If the most serious option(s) checked above are Minimal risk, then this is a Minimal^m^ risk adverse event.				

Additional details (including “other” entries):

Footnotes:

^a^“Subclinical respiratory depression” is defined as capnographic abnormalities suggesting respiratory depression that do not manifest clinically

^b^“Paradoxical response” is defined as unanticipated restlessness or agitation in response to sedatives

^c^“Recovery agitation” is defined as abnormal patient affect or behaviors during the recovery phase that can include crying, agitation, delirium, dysphoria, hallucinations, or nightmares

^d^“Prolonged recovery” is defined as failure to return to baseline clinical status within 2 h

^e^“Failed sedation” is defined as inability to attain suitable conditions to humanely perform the procedure

^f^Alteration in vital signs (bradycardia, tachycardia, hypotension, hypertension) is defined as a change of >25% from baseline.

^g^“Cardiovascular collapse/shock” is defined as clinical evidence of inadequate perfusion

^h^Examples of “escalation of care” include transfer from ward to intensive care, and prolonged hospitalization

^i^“Pulmonary aspiration syndrome” is defined as known or suspected inhalation of foreign material such as gastric contents into the respiratory tract associated with new or worsening respiratory signs

^j^“Sentinel” adverse events are those critical enough to represent real or serious imminent risk of serious and major patient injury. Once recognized, they warrant immediate and aggressive rescue interventions. Once clinically concluded, they warrant immediate reporting within sedation care systems, and the highest level of peer scrutiny for continuous quality improvement

^k^“Moderate” adverse events are those that, while not sentinel, are serious enough to quickly endanger the patient if not promptly managed. Once clinically concluded, they warrant timely reporting within sedation care systems, and periodic peer scrutiny for continuous quality improvement

^l^“Minor” adverse events are those encountered periodically in most sedation settings and that pose little threat given appropriate sedationist skills and monitoring.

^m^“Minimal” adverse events are those that alone present no danger of permanent harm to the patient

All participants will receive 10 g of 2% lidocaine hydrochloride mucilage. They will be administered supplemental oxygen (3–4 L/min) before injecting sufentanil until the participants are fully alert after the procedure. Approximately 1 min after administering 5 μg of sufentanil, remimazolam tosylate 0.15 mg/kg, remimazolam tosylate 0.2 mg/kg, or propofol 1.5 mg/kg will be used to induce sedation. If the MOAA/S score is ≤2, upper gastrointestinal endoscope intubation will be initiated. If the MOAA/S score is still >2 after two min or if the upper gastrointestinal endoscopy attempt fails because the participant is writhing or nauseating, the experimental group will be given remimazolam tosylate (0.05 mg/kg) and the control group will be given propofol (0.5 mg/kg). The administration time is 10 s, and the interval between each supplementation dose is at least 1 min. After the endoscope intubation, to maintain a certain degree of sedation (MOAA/S score, ≤2), additional remimazolam tosylate or propofol will be administered as necessary. If two doses (after the initial dose) within any 5-min window are not sufficient to obtain or maintain adequate sedation, the case will be designated a treatment failure and rescue sedative medication (propofol) will be administered to obtain or maintain adequate sedation to complete upper gastrointestinal endoscopy.

Participants may have perioperative respiratory-related adverse events during upper gastrointestinal endoscopy. If the SpO_2_ is <95%, we will lift the patient’s mandible. If it is <90%, we will increase the oxygen flow of the nasal catheter to 6 L/min. If the SpO_2_ is still <90%, we will suspend the procedure, pull out the upper gastrointestinal endoscope, and perform positive pressure ventilation with a mask. If it still cannot be corrected, laryngeal mask airway or tracheal intubation will be performed to control breathing. During the procedure, participants with hypotension or bradycardia will be administered ephedrine or atropine, as appropriate.

#### Interventions, modifications, adherence, and concomitant care

The assigned intervention will be discontinued only in response to participant’s request. No intervention modification is planned during the trial. Adherence to interventions mainly refers to participant self-management adherence. No concomitant care or interventions are permitted during this trial.

#### Plans for collection, laboratory evaluation, and storage of biological specimens for genetic or molecular analysis in this trial/future use

We have no plans to collect or store biological specimens involved in this trial.

#### Outcome measures

Primary outcome measures

The primary outcome is the success rate of remimazolam tosylate for sedation for diagnosis and treatment during upper gastrointestinal endoscopy. Success is defined by a composite measure (all three conditions must be met) of the following:

1. Completion of the procedure of upper gastrointestinal endoscopy.
2. No requirement for rescue sedative medication.
3. After administering the initial dose of the trial drug, additional administration is ≤2 times within any 5-min period.

Secondary outcome measures

The secondary outcomes are as follows:

1. The induction time of sedation: defined as the time interval from the initial administration of the trial drug to the first MOAA/S score of ≤2.
2. The time of full alertness: defined as the time from the discontinuation of sedative medication to full alertness (the first of three consecutive MOAA/S scores of 5).
3. The incidence of drug injection pain.
4. Digestive endoscopist and participant satisfaction, measured on a scale from 1 to 10. Endoscopists and participants will score their satisfaction with the scale, with 1–3 (dissatisfied), 4–6 (satisfied), or 7–10 (very satisfied), after upper gastrointestinal endoscopy.
5. The time of discharge: defined as the time interval from the termination of the administration of the trial drug to meeting the discharge criteria (modified postanesthesia discharge scoring system score of ≥9, with two points in the vital sign item).
6. The incidence of hypoxia during sedation: defined as 75% ≤ SpO_2_ < 90% for <60 s.
7. All adverse events occurring during the procedure were recorded using tools proposed by the World Society of Intravenous Anesthesia (SIVA)’s International Sedation Task Force [19].

#### Participant timeline

**Fig. 1:**
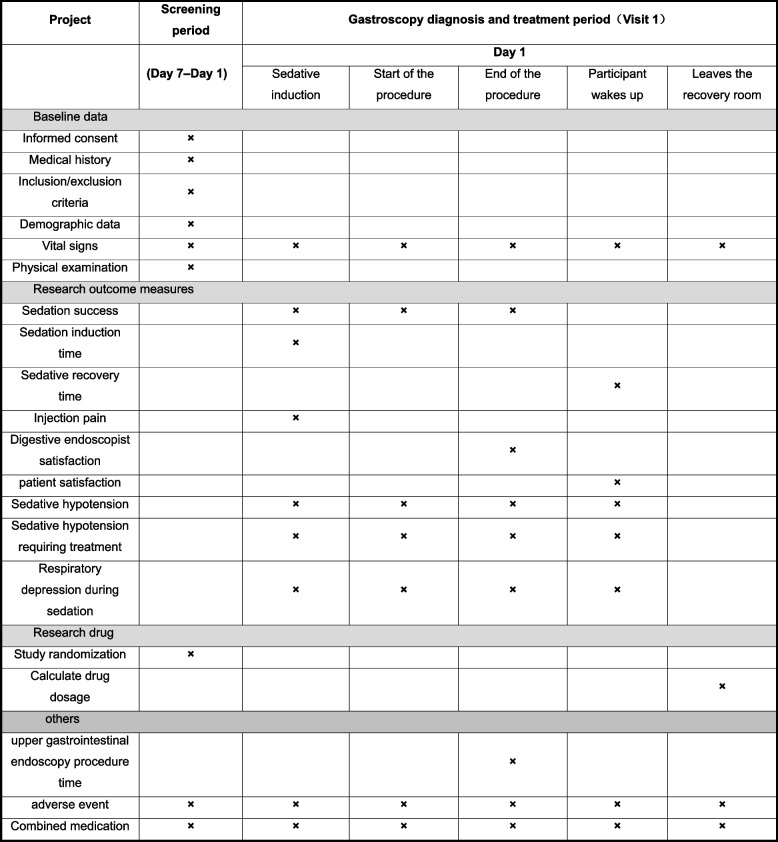
Schedule of the major study events

Figure 1 presents the schedule for enrollment, evaluation of research outcome measures, and others for participants.

We will perform upper gastrointestinal endoscopic sedation on the first day, and the baseline data of participants will be collected and randomized before conducting the procedure. Participants’ vital signs will be monitored. Adverse events will be assessed, and drug combination will be recorded during the whole procedure.

#### Sample size

In this study, the noninferiority test will be performed between the experimental and control groups (i.e., experimental group 1 vs. control group; experimental group 2 vs. control group). Assume that the success rate of sedation in the propofol group is 100% and that in each remimazolam tosylate group is 95%. Considering the first type of error, *α* = 0.0125 (single-sided), 90% power, and 10% dropout rate, 600 participants are required for each group, with a total of 1800 for the three groups.

#### Allocation masking

This study follows a single-blinded method. All participants are blinded to the assignment of the trial (the injection site will be shielded) and will be randomly assigned to any of the three groups.

#### Unblinding procedures

Because the trial is single blind, participants interested in knowing their group can be informed by the investigator after analyzing the results.

#### Plans for communicating important protocol modifications to relevant parties

All changes in the study protocol will be reviewed by the ethical committee, which will be reported to the sponsor, participating care providers, and investigators.

#### Composition of the data-monitoring committee and its role and reporting structure

We have not considered the need for a data-monitoring committee.

#### Interim analyses

No formal interim analysis of the primary and secondary outcomes is planned.

#### Frequency and plans for auditing trial conduct

We have no plans for auditing trial conduct in this investigator-initiated pragmatic trial.

#### Data management

All participants’ data collected during this clinical study will be entered and/or filed in the respective participants’ case report forms (CRFs). Data on each patient’s study participation must be documented appropriately in the CRF with study number, participant number, date of collection of participant information, and informed consent. The source data should be filed based on the GCP guidelines. The data manager will be responsible for data processing based on the sponsor’s standard operating procedures and will conduct regular monitoring to ensure that dates are adequate, accurate, and complete. Database lock will occur only after completing the quality assurance procedures.

#### Statistical analysis

Data selection for statistical analysis

1. Full analysis set (FAS): Based on the principle of intention-to-treat analysis, the full analysis set will include all participants who enroll in the study and receive at least one dose of the treatment.
2. Per-protocol set (PPS): The PPS population will include all FAS participants without major protocol deviations that influence the evaluation of the primary outcomes. Efficacy analysis will be performed on the FAS and PPS.
3. Safety analysis set (SAS): The safety population consists of all participants who receive at least one dose of the treatment. Analyses of the safety data in this study will be based on the safety population.

##### Statistical analysis plan

All statistical analyses in this experiment will be programmed and calculated using the SAS analytics software version 9.4. In this study, the noninferiority one-sided test used for the main efficacy outcome is that a *P*-value of ≤0.0125 will be considered statistically significant. All other tests will be two-sided tests, with a *P*-value of ≤0.05 considered statistically significant, and the confidence interval will be set at 95%.

Measurement of basic data in different treatment groups will be statistically expressed as mean ± standard deviation or median (interquartile range [IQR]).

The *χ*^2^ test will be used for the success rate of sedation, incidence of injection pain, hypoxia, and adverse events. The analysis of variance will be used to induce time of sedation, full alertness, and discharge. The rank sum test will be used for calculating the satisfaction of endoscopists and participants.

##### Additional analyses

1. Subgroup analysis: Subgroup analysis via an interaction by gender, age (18–24, 25–44, and 45–60 years), and BMI (<23 and ≥23 kg/m^2^) will be used to analyze the primary and secondary outcomes among subgroups.
2. Safety analysis: General safety evaluations will be based on the incidence and type of adverse events. Safety variables will be tabulated and presented for all participants in the safety set. Adverse events will be coded using the World SIVA adverse sedation event reporting tool. The number (%) of participants with any adverse events will be summarized.

#### Methods to handle protocol nonadherence and statistical methods to handle missing data

Statistical analysis will be conducted on an intention-to-treat basis. The outcomes will be analyzed as randomized, regardless of protocol adherence. All variables will be screened for frequency and type of missingness. Multiple imputation will be used if missingness is >5% in any variable. In the case of missing data and imputation, complete case analysis will be performed as a sensitivity analysis.

#### Composition of the coordinating center and trial steering committee

Diansan Su (DSS), the principal investigator, is responsible for preparing and revising the protocol and disseminating any changes. Huichen Zhu (HCZ) and Zhongxue Su (ZXS) are responsible for coordinating data collection and analyses and for writing the scientific manuscript. Xiaorong Huai (XRH), a senior investigator, is responsible for overseeing the study design and protocol and for the interpretation of the findings. Caiyang Chen (CYC), a statistician, is responsible for overseeing any statistical analyses. Jie Zhou (JZ), a clinical investigator, is responsible for overseeing that the study implementation on the floor follows the protocol.

#### Dissemination plans

The study results will be disseminated through articles published in peer-reviewed journals.

#### Additional consent provisions for the collection and use of participant data and biological specimens

No ancillary study is planned, and no plans are in place to collect additional participant data or biological specimens outside of what is mentioned in this protocol.

#### Adverse event reporting and harm

Remimazolam tosylate, a new rapidly metabolizing benzodiazepine, may cause some minor adverse effects but is generally well tolerated. We will evaluate any harm from the intervention. There is a commentary section in study-specific CRFs where researchers can report allocation violations or any unexpected side effects from the allocated intervention. We will collect all expected and unexpected drug-related adverse events nonsystematically and will report all harm in trial publications.

## Discussion

Our previous study demonstrated that most patients (85.4%) felt uncomfortable during unsedated gastroscopy and 2.1% of the patients reported extreme discomfort. Vomiting/retching was the most common adverse event reported during unsedated gastroscopy. A larger number of gastroscope-intubation attempts and a longer procedure time were needed for unsedated gastroscopy. Therefore, sedative gastroscopy is essential for patients [20]. However, the sedation rate for gastrointestinal endoscopy is much lower in China than in the USA and Europe [21].

Significant developments in endoscopic sedatives have been made in the past decade with participant safety and comfort being the primary focus. However, sedation increases the costs and poses a risk for complications [22].

In the past, propofol and midazolam were the main drugs used for gastrointestinal endoscopic sedation [1]. Propofol is the preferred medication, especially by anesthesiologists. However, propofol use is usually associated with hemodynamic suppression [23]. It is a very negative inotropic drug wherein any decrease in the cardiac output cannot be compensated by an increase in the heart rate. Furthermore, propofol inhibits the sympathetic tone, indicating that cardiac output often drops significantly after a bolus dose. This is most pronounced in patients with impaired cardiac function, especially the elderly and neonates. Propofol induces dose-dependent respiratory depression, whose incidence and duration depend on age, dose, rate of injection, and co-administration of other sedatives/analgesics [24].

Midazolam is the most commonly used sedative drug because of the legal restriction of propofol. Midazolam has a slower onset of action and a relatively longer half-life time (1 h). It is metabolized into active metabolites and interacts with all drugs metabolized through the cytochrome P450 pathway [25]. Furthermore, the creation of active metabolites may lead to a longer and less predictable recovery from sedation [8]. Considering the increasing demand for gastrointestinal endoscopy and disadvantages of existing anesthetics, the development of more effective agents with a more tolerable profile is required urgently.

Remimazolam is a new and rapidly metabolizing benzodiazepine drug [26] produced from a method developed for “soft” drugs to form compounds by fusing known sedative hypnotic drugs with metabolically unstable parts (most commonly esters) to promote predictable and rapid enzyme metabolism, thereby increasing hemodynamic stability and reducing adverse reactions associated with the parent compound [27, 28]. After the resulting structural modification, it undergoes organ-independent metabolism by tissue esterases into an inactive metabolite [29]. Remimazolam is rapidly hydrolyzed to carboxylic acid CNS 7054 [10], and its in vitro affinity for human GABAA receptors is 400 times lower than that of midazolam [14]. Thus, the metabolite is not expected to produce important behavioral effects. Animal studies have confirmed that remimazolam is a potent, rapidly metabolizing hypnotic with a significantly shorter duration of action than that of midazolam. In mice, approximately equihypnotic doses of remimazolam and midazolam produced loss of righting reflexes for durations of several minutes and nearly an hour, respectively [10]. Remimazolam produces modest respiratory and cardiovascular depression at sedating doses, a side effect profile shared with midazolam and other benzodiazepines [26, 30]. Studies have shown that remimazolam has a sedative effect in sheep, and a dose of 0.37–2.21 mg/kg can produce a short-term sedative effect without causing excessive respiratory or cardiovascular depression [26]. Furthermore, comparing the advantages and disadvantages of remimazolam with those of midazolam and propofol in sheep, remimazolam has a rapid offset and greater depth of sedation compared with those of midazolam [30]. In a phase I clinical study, compared with midazolam, remimazolam showed a rapid onset, could be offset, was well tolerated, and had basically stable hemodynamics [14]. Remimazolam had a higher success rate [31], and the waking up time was shorter [32] compared with that of midazolam. Other clinical studies have also reported similar results [33, 34]. Preliminary phase II clinical trials have also shown minimal residual effects of remimazolam in long-term infusions [35]. Studies have shown that remimazolam can effectively replace propofol in gastrointestinal endoscopic sedation without compromising the quality of sedation [36]. In a multicenter, single-blind, randomized, parallel-group phase IIb/III trial, remimazolam was well tolerated as a sedative and hypnotic for general anesthesia and was not inferior to propofol [37]. Based on the abovementioned advantages, remimazolam is very suitable for anesthesia for procedures such as bronchoscopy and gastrointestinal endoscopy. Studies have shown that in bronchoscopy, remimazolam has an effective sedative effect, a medication with rapid onset and recovery compared with midazolam [38]. Phase I–III studies of gastrointestinal endoscopy have also shown that remimazolam presented sufficient sedation, rapid recovery, and rapid recovery of the nerve function compared with midazolam [13, 39–41]. Compared with propofol, remimazolam use has less injection pain and hypotension, characterized by a strong candidate replacement drug for propofol and midazolam in gastrointestinal endoscopy anesthesia [37].

Remimazolam tosylate is a new short-acting GABAA receptor agonist developed by HengRui Medicine Co., Ltd., China. It has been approved by the National Medical Products Administration as a new anesthetic and sedative in 2019 [42]. Remimazolam tosylate has been investigated as a novel ultra-short-acting benzodiazepine in a phase III clinical trial for procedural sedation in China [43]. The study showed that a single dose of remimazolam tosylate (5.0 mg), followed by top-up doses as necessary, was noninferior to propofol in providing adequate sedation, with a very high success rate for patients undergoing upper gastrointestinal endoscopy. The initial low dose of 5.0 mg for remimazolam tosylate may not sufficiently induce rapid sedation. Thus, investigations on the optimized initial loading dose remain to be warranted.

Based on previous studies [44–46], we administered fixed doses of sufentanil. Fixed-dose administration is convenient and more in line with clinical practice.

Based on the existing data, remimazolam tosylate is promising to be used as a sedative agent for outpatient procedural sedation where predictable and rapid recovery is highly desirable. However, no clear standard has been established for the current dosage. Excessive use can cause respiratory depression, a fatal condition when involving personnel without professional training in airway management. Furthermore, if the dosage is insufficient, the participant cannot achieve ideal sedation. Further studies on human subjects are needed to completely characterize its action, which will define the optimal dosing regimens and to determine whether metabolite accumulation with prolonged infusion delays recovery.

## Trial status

This trial was registered on 18 February 2021 in ClinicalTrials.gov, NCT04727034.

The protocol version is 1.1, which was approved in January 2021. The experiment is expected to start on February 8, 2021. The five centers are expected to complete the evaluation of 1800 cases by December 31, 2022. Data analysis and publication of the paper are expected to be completed by July 1, 2023.

## Supplementary Information

**Additional file 1.** SPIRIT checklist.

**Additional file 2.** Supplemental table.

## Data Availability

Data on participants cannot be made publicly available because of the Chinese data protection rules and regulations. The statistical code will be available upon request.

## Abbreviations

MOAA/S: Modified Observer’s Assessment of Alertness/Sedation
GABAA: Type A gamma-aminobutyric acid
ASA: American Society of Anesthesiologists
BMI: Body mass index
ULN: Upper limits of normal
GCP: Good Clinical Practice
SpO_2_: Peripheral oxygen saturation
CPAP: Continuous positive airway pressure
SIVA: Society of Intravenous Anesthesia
CRFs: Case report forms
FAS: Full analysis set
PPS: Per-protocol set
SAS: Safety analysis set
IQR: Interquartile range
