# Supplementary material for: Efficacy and safety of remimazolam tosylate for sedation during upper gastrointestinal endoscopy: study protocol for a multicenter randomized controlled trial
Source: Trials. 2022 Dec 12;23:995. doi: 10.1186/s13063-022-06935-0 (PMC9743763; doi:10.1186/s13063-022-06935-0)
Supplement: Supplementary file 2 — Additional file 2. Supplemental table. [file 13063_2022_6935_MOESM2_ESM.docx]

Supplemental Table：Items from the World Health Organizaion Trial Registration Data Set

| 1. Primary registry and trial-identifying number: ClinicalTrials.gov, NCT04727034 2. Date of registration in primary registry: 18 February 2021 3. Secondary indentifying numbers: no 4. Sources of monetary or material support: no 5. Primary sponsor: no 6. Secondary sponsor(s): no 7. Contace for public queries: MD Huichen Zhu, department of anesthesiology, Renji Hospital, Shanghai Jiaotong University School of Medicine 8. Contace for scientific queries: PHD. Diansan Su, department of anesthesiology, Renji Hospital, Shanghai Jiaotong University School of Medicine 9. Publice title: Efficacy and safety of remimazolam tosylate for sedation during upper gastrointestinal endoscopy: Study protocol for a multi-centered randomized controlled trial 10. Scientific title: see 9 11. Countries of recruitment: China, Shanghai；Nanchang；Jiaxing 12. Health condition(s) or problem(s) studied: sedation for upper gastrointestinal endoscopy 13. Intervention(s): perioperative intervention for sedation during upper gastrointestinal endoscopy 14. Key inclusion criteria: Age, ≤18 and ≤60 years, no gender limit；Undergoing routine upper gastrointestinal endoscopic diagnosis and treatment；ASA classification I–II；18 kg/m^2^ < body mass index (BMI) < 28 kg/m^2^；Time of upper gastrointestinal endoscopy not exceeding 30 min；Clearly understand and voluntarily participate in the study; provide signed informed consent   Key exclusion criteria: 1) Need to perform complicated endoscopic techniques for diagnosis and treatment, such as cholangiopancreatography surgery, endoscopic ultrasonography, endoscopic mucosal resection, endoscopic submucosa stripping, and oral endoscopic muscle dissection；2) Intend to undergo tracheal intubation；3) Judged to have difficulty in managing the respiratory tract (modified Mallampati score is IV)；4) Anemia or thrombocytopenia, (hemoglobin < 90 g/L, platelet count <80 × 10^9^/L)；5) Diagnosed with lung diseases (asthma, bronchitis, chronic obstructive pulmonary diseases, pulmonary bullae, pulmonary embolism, pulmonary edema, and lung cancer)；6) Diagnosed with liver and kidney diseases (aspartate aminotransferase and/or alanine aminotransferase ≥2.5 × upper limits of normal (ULN), total bilirubin ≥1.5 × ULN, and blood creatinine levels greater than the upper normal limit)；7) History of drug and/or alcohol abuse within 2 years before initiating the screening period; average daily alcohol consumption of >2 units of alcohol (1 unit = 360 mL beer or 45 mL liquor with 40% alcohol content or 150 mL grapes liquor)；8) Blood pressure not satisfactorily controlled by antihypertensive drugs (sitting systolic blood pressure, ≥160 mmHg during the screening period and/or diastolic pressure during the screening period pressure, ≥100 mmHg)；9) Sitting systolic blood pressure of ≤90 mmHg during the screening period；10) Pregnant or breastfeeding；11) Allergies or contraindication to benzodiazepines, opioids, propofol, and lidocaine；12) Participated in other drug clinical trials in the past 3 months；13) Investigator’s judgment as an unsuitable participant；14) Diagnosed with heart disease (heart failure, angina pectoris, myocardial infarction, and heart rhythm abnormalities)   1. Study type: multicentre randomised controlled trial 2. Date of first enrolment: 2 March 2021 3. Target sample size: 360 4. Recruitment status: enrolling by invitation 5. Primary outcome(s): t1) Completion of the procedure of upper gastrointestinal endoscopy. 2) No requirement for rescue sedative medication.3) After administering the initial dose of the trial drug, additional administration is ≤2 times within any 5-min period. 6. Key secondary outcome(s): 1) The induction time of sedation: defined as the time interval from the initial administration of the trial drug to the first MOAA/S score of ≤2.2) The time of full alertness: defined as the time from the discontinuation of sedative medication to full alertness (the first of three consecutive MOAA/S scores of 5).3) The incidence of drug injection pain.4) Digestive endoscopist and participant satisfaction, measured on a scale from 1 to 10. Endoscopists and participants will score their satisfaction with the scale, with 1–3 (dissatisfied), 4–6 (satisfied) or 7–10 (very satisfied) after upper gastrointestinal endoscopy.5) The time of discharge: defined as the time interval from the termination of administration of the trial drug to meeting the discharge criteria (modified post-anesthesia discharge scoring system score of ≥9, with 2 points in the vital sign item).6) The incidence of hypoxia during sedation: defined as 75% ≤ SpO2 < 90% for <60 s, SpO2 <75% for any duration or 75% < SpO2 < 90% for ≥ 60 s).7) All adverse events occurring during the procedure were recorded using tools proposed by the World Society of Intravenous Anesthesia (SIVA)’s International Sedation Task Force. |
| --- |

Supplemental Table：Items from the World Health Organizaion Trial Registration Data Set
